# Biomaterials promote *in vivo* generation and immunotherapy of CAR-T cells

**DOI:** 10.3389/fimmu.2023.1165576

**Published:** 2023-04-20

**Authors:** Ya-Ting Qin, Ya-Ping Li, Xi-Wen He, Xi Wang, Wen-You Li, Yu-Kui Zhang

**Affiliations:** ^1^ College of Chemical and Biological Engineering, Zhejiang University, Hangzhou, China; ^2^ ZJU-Hangzhou Global Scientific and Technological Innovation Center, Zhejiang University, Hangzhou, China; ^3^ State Key Laboratory of Medicinal Chemical Biology, Tianjin Key Laboratory of Biosensing and Molecular Recognition, Research Center for Analytical Sciences, College of Chemistry, Nankai University, Tianjin, China; ^4^ National Chromatographic Research and Analysis Center, Dalian Institute of Chemical Physics, Chinese Academy of Sciences, Dalian, China

**Keywords:** biomaterials, immunotherapy, gene delivery, solid tumor treatment, chimeric antigen receptor-T

## Abstract

Chimeric antigen receptor-T (CAR-T) cell therapy based on functional immune cell transfer is showing a booming situation. However, complex manufacturing processes, high costs, and disappointing results in the treatment of solid tumors have limited its use. Encouragingly, it has facilitated the development of new strategies that fuse immunology, cell biology, and biomaterials to overcome these obstacles. In recent years, CAR-T engineering assisted by properly designed biomaterials has improved therapeutic efficacy and reduced side effects, providing a sustainable strategy for improving cancer immunotherapy. At the same time, the low cost and diversity of biomaterials also offer the possibility of industrial production and commercialization. Here, we summarize the role of biomaterials as gene delivery vehicles in the generation of CAR-T cells and highlight the advantages of *in-situ* construction *in vivo*. Then, we focused on how biomaterials can be combined with CAR-T cells to better enable synergistic immunotherapy in the treatment of solid tumors. Finally, we describe biomaterials’ potential challenges and prospects in CAR-T therapy. This review aims to provide a detailed overview of biomaterial-based CAR-T tumor immunotherapy to help investigators reference and customize biomaterials for CAR-T therapy to improve the efficacy of immunotherapy.

## 1 Introduction

The treatment of malignant tumors is still a worldwide critical topic. In the recent decade, immunotherapy has boosted and shown significant advantages in cancer treatment (1). Unlike conventional cancer treatment (e.g. surgery, chemotherapy, radiotherapy) (2), immunotherapy relies on activated immune cells to recognize and attack specific tumor cells that avoid severe side effects (3). Currently, adoptive cell therapy, also known as tumor immune cell therapy is one of the main strategies in cancer immunotherapy, which extracts immune cells from the body, amplifies the desirable immune cells, and injects them back into the body to trigger the immune effect on tumor cells (4). Clinical studies have shown that patients with malignant tumors usually retain several tumor cells in the body after surgery, radiotherapy, and chemotherapy, which is the main reason leading to the recurrence after treatment (5). In fact, cellular immunotherapy can stimulate the immune system to kill the residual tumor cells in the body and prevent tumor metastasis and recurrence. However, early cell therapies are non-specific that largely limit the efficacy, such as insufficient recognition, tumor immune escape, loss of major histocompatibility complex (MHC), and immune tolerance of host cells (6).

**Figure 1:**
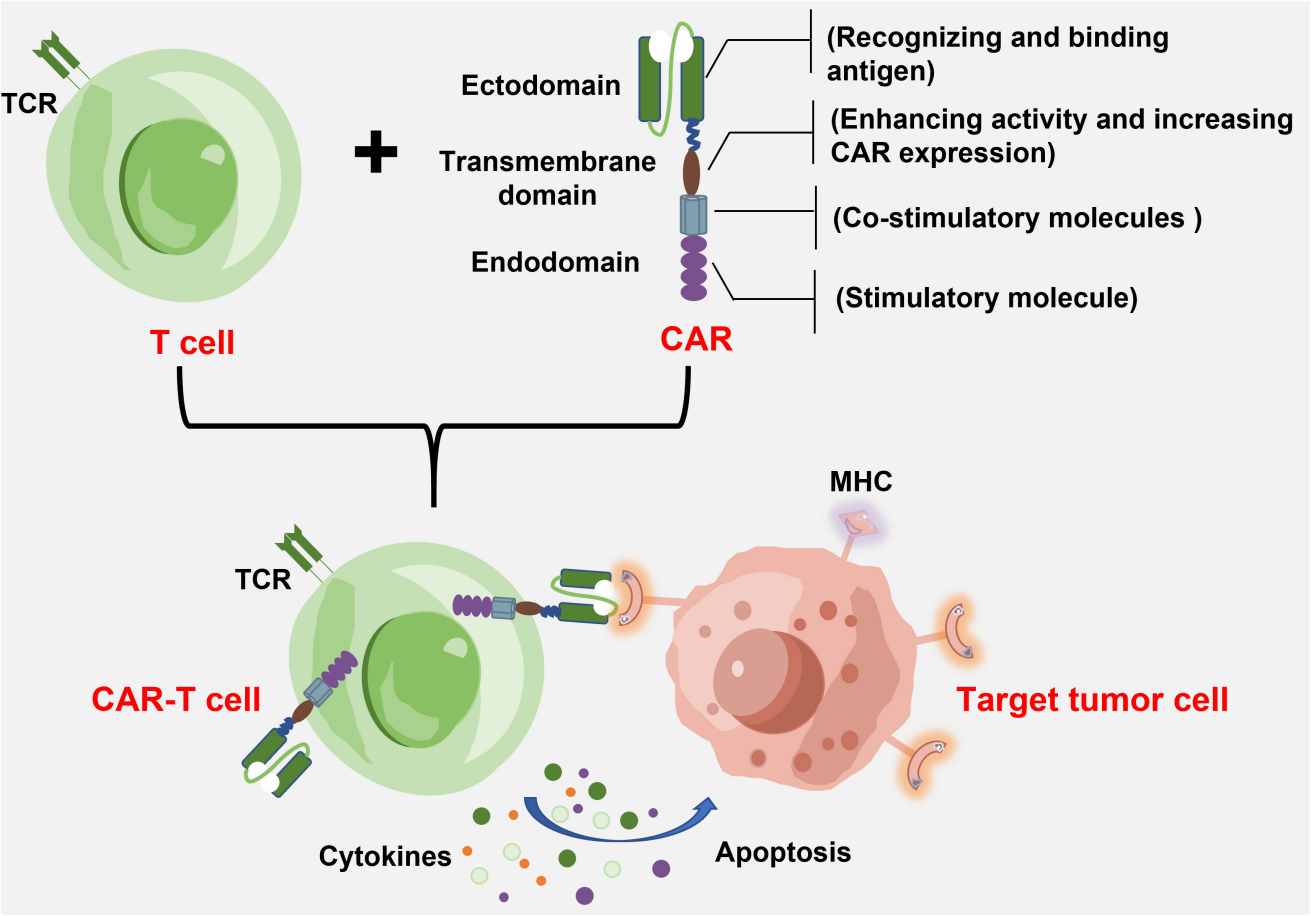
The basic structure of CAR-T cell and target tumor cell.

Chimeric antigen receptor-T immunotherapy is to engineer a patient’s autologous T cells to express the antigen receptor, which specifically recognizes antigen molecules of the tumor cell surfaces (7). As shown in **Figure 1**, the basic structure of CAR consists of an extracellular antigen recognition domain (to recognize and bind antigens), a transmembrane domain (to enhance activity and increase CAR expression), and an intracellular signal transduction domain (for co-stimulation and signal transduction) (8). The extracellular antigen recognition domain is composed of a single-chain variable fragment (scFv), which specifically recognizes tumor-associated antigens or tumor-specific antigens and determines the tumor targeting of a CAR-T cell. When a T cell recognizes the tumor antigen, the binding of scFv segments to tumor antigens activates and initiates the T-cell killing mechanism (9). Unlike natural T-cell receptors that rely on MHC presentation mechanisms, the CAR structure enables T cells to directly target tumor cell surface antigens toward the specific killing of tumors, which avoids tumor immune escape caused by MHC molecular restriction and downregulation (10). In addition, CAR also recognizes and binds polysaccharides and lipid proteins on the cell surface due to the antigen-antibody mechanism. When CAR-T cells are re-infused into the body, they recognize tumor cells specifically and can be activated. These activated CAR-T cells proliferate consistently to maintain lethality while releasing cytokines that activate other immune cells collaboratively against the tumor (11). Thus, a CAR-T cell contains both merits of the high targeting specificity of antigens and the cytotoxicity of T cells against the target tumor (12). The CAR-T therapy shows strong lethality and targeting tumors, and longer-lasting efficacy compared with other treatments.

So far, CAR-T therapy has achieved considerable efficacy in treating various hematological tumors (13). Since the approval of Kymirah, a CAR-T cell drug developed by Novartis in 2017, seven more drugs have been approved, providing unprecedented therapeutic outcomes for patients with a variety of hematologic tumors (**Table 1**). While the remarkable efficacy of CAR-T therapy attracted wide attention from both academic and clinical researchers, the therapy has shown some limitations and great challenges with in-depth research and applications. For example, since the lentivirus and retrovirus are used as the vector of the CAR genes, it may cause safety issues (14). For solid tumors, the complex tumor microenvironment inhibits intratumoral invasion of CAR-T cells, as well as the proliferation and activation of lymphocytes infiltrating into the tumor (15). Also, the high cost of producing CAR-T cells *in vitro* might restrict its broad applications. Therefore, researchers are dedicated to developing novel approaches to overcome the above obstacles during CAR-T preparation and improve the *in vivo* application.

**Table 1:** CAR-T cell therapies currently on the market.

Name	Target	Manufacturer	Indication	Price/needle	Country	First approval	Carrier
Kymriah (tisagenlecleucel)	CD19	Novartis Pharmaceuticals Corporation	Relapsed or refractory follicular lymphoma	47.5 thousand dollars	USA(2017)/EU(2018)/Cananda(2019)/Janpan(2019)	2017-09-30	Lentivirus
Yescarta (axicabtagene ciloleucel)	CD19	Kite Pharma Inc.	Large B-cell lymphoma	37.5 thousand dollars	USA(2017)/EU(2018)/Cananda(2019)	2017-10-18	Retrovirus
Tecartus (brexucabtagene autoleucel)	CD19	Kite Pharma, Inc.	Relapsed or refractory mantle cell lymphoma, relapsed or refractory (r/r) B-cell, precursor acute lymphoblastic leukemia	37.3 thousand dollars	USA(2020)/EU(2020)	2020-10-28	Retrovirus
Breyanzi (lisocabtagene maraleucel)	CD19	Juno Therapeutics, Inc., a Bristol-Myers Squibb Company	Large B-cell lymphoma, diffuse large B-cell lymphoma, high-grade B-cell lymphoma, primary mediastinal large B-cell lymphoma, and follicular lymphoma grade 3B	41.03 thousand dollars	USA(2021)	2021-02-05	Lentivirus
Abecma (idecabtagene vicleucel)	BCMA	Celgene Corporation, a Bristol-Myers Squibb Company	Relapsed or refractory multiple myeloma	43.7968 thousand dollars	USA(2021)	2021-03-26	Lentivirus
Yescarta (AxicabtageneCiloleucel)	CD19	Fosunkitebio.	Relapsed or refractory (r/r) B-cell	1.2 million RMB	CN(2021)	2021-06-22	Retrovirus
Relma-cel (Relmacabtagene autoleucel)	CD19	JW (Cayman) Therapeutics Co. Ltd	Relapsed or refractory (r/r) B-cell	Unknown	CN(2021)	2021-09-03	Unknown

In recent years, biomaterials have attracted growing interest in the field of tumor immunotherapy due to their significant biocompatibility, targeting, and controllable release (16). Applying biomaterials to CAR-T therapy presents new opportunities to overcome the challenges it encountered, such as the unsafety of viral vehicles, poor efficacy in solid tumor therapy, and complex and costly producing processes. For instance, many nanomaterials are used as vehicles to deliver CAR-expressing genes to T cells *in vivo* and *in vitro* to avoid the safety concerns of viruses. In the treatment of solid tumors, biomaterials (e.g. nanoparticles, hydrogels, and biological scaffolds) can be used as vehicles for drugs, active biomolecules, and therapeutic cells. This can promote the transport of CAR-T cells, improve the expansion and infiltration of immune cells, and amplify the immunomodulatory effect through synergistic therapeutic effects. It should be emphasized that nanomaterials can directly deliver CAR-expressing genes into circulating T cells *in vivo* to achieve *in situ* generations of CAR-T cells, which significantly simplifies the process of CAR-T preparation and treatment. Compared to CAR-T therapy, the preparation of biomaterials is simple and not expensive, which is promising to be used to optimize the process of CAR-T therapy (17).

In this paper, we systematically review how biomaterials can engineer CAR-T cells in cancer therapy to improve their immune function and offer a prospect for their development. First, we outline the advantages of nanomaterials as gene vehicles for the more efficient construction of CAR-T cells, with particular emphasis on their *in-situ* generation *in vivo*. Compared with viral vehicles, nanomaterials are more flexible and safer. Second, we mainly summarize how multiple forms of biomaterials loaded with multiple functional biomolecules bind to CAR-T cells to promote synergistic immunotherapy in solid tumors. Finally, the potential challenges and future research directions of biomaterial-based CAR-T therapy in tumor treatment were identified. It is expected to provide readers with an overview of biomaterial-based CAR-T therapy to facilitate the application of biomaterials in CAR-T therapy.

## 2 Biomaterials promote the generation of CAR-T cells

**Figure 2:**
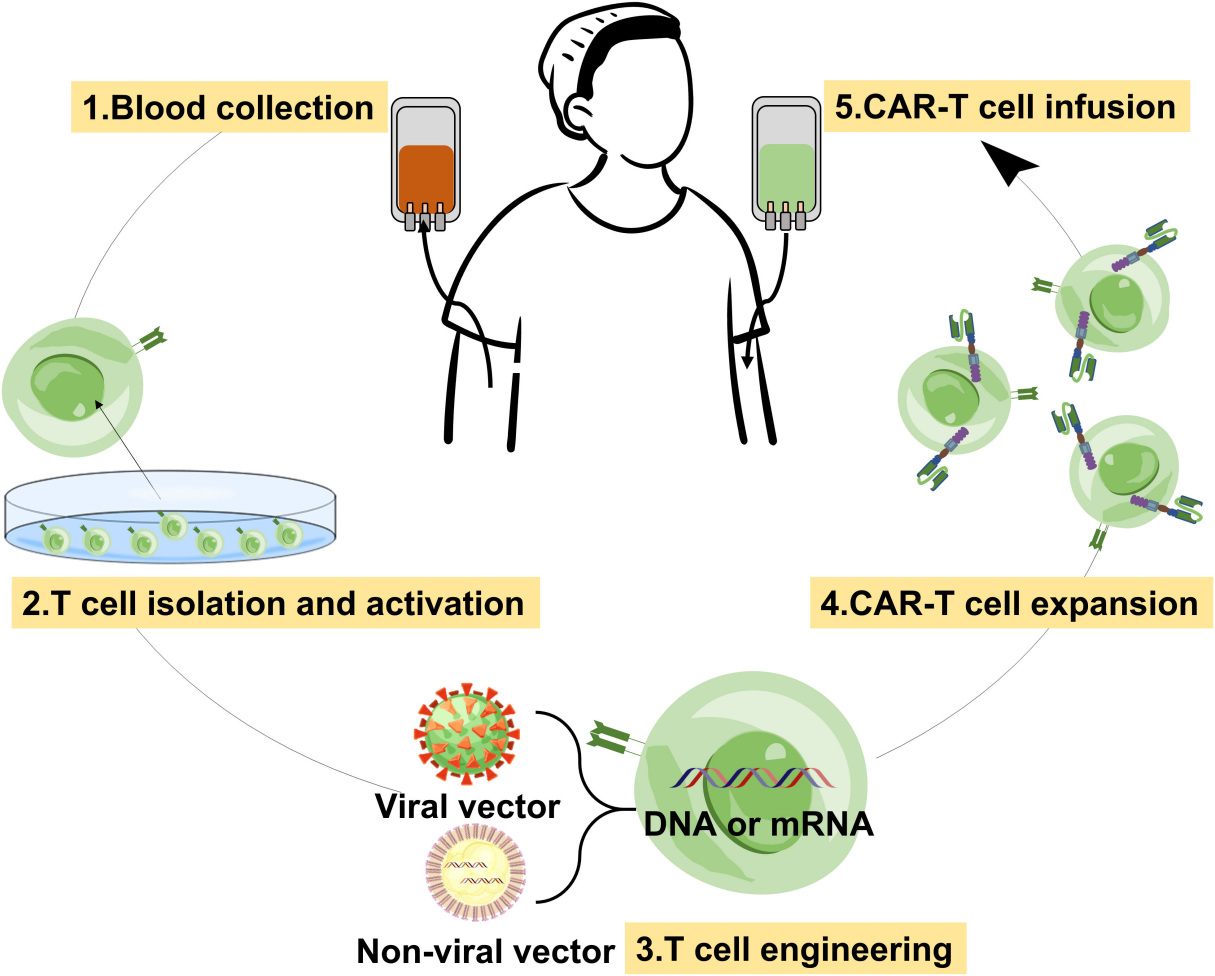
Schematic diagram of the process of conventional CAR-T therapy.

**Figure 3:**
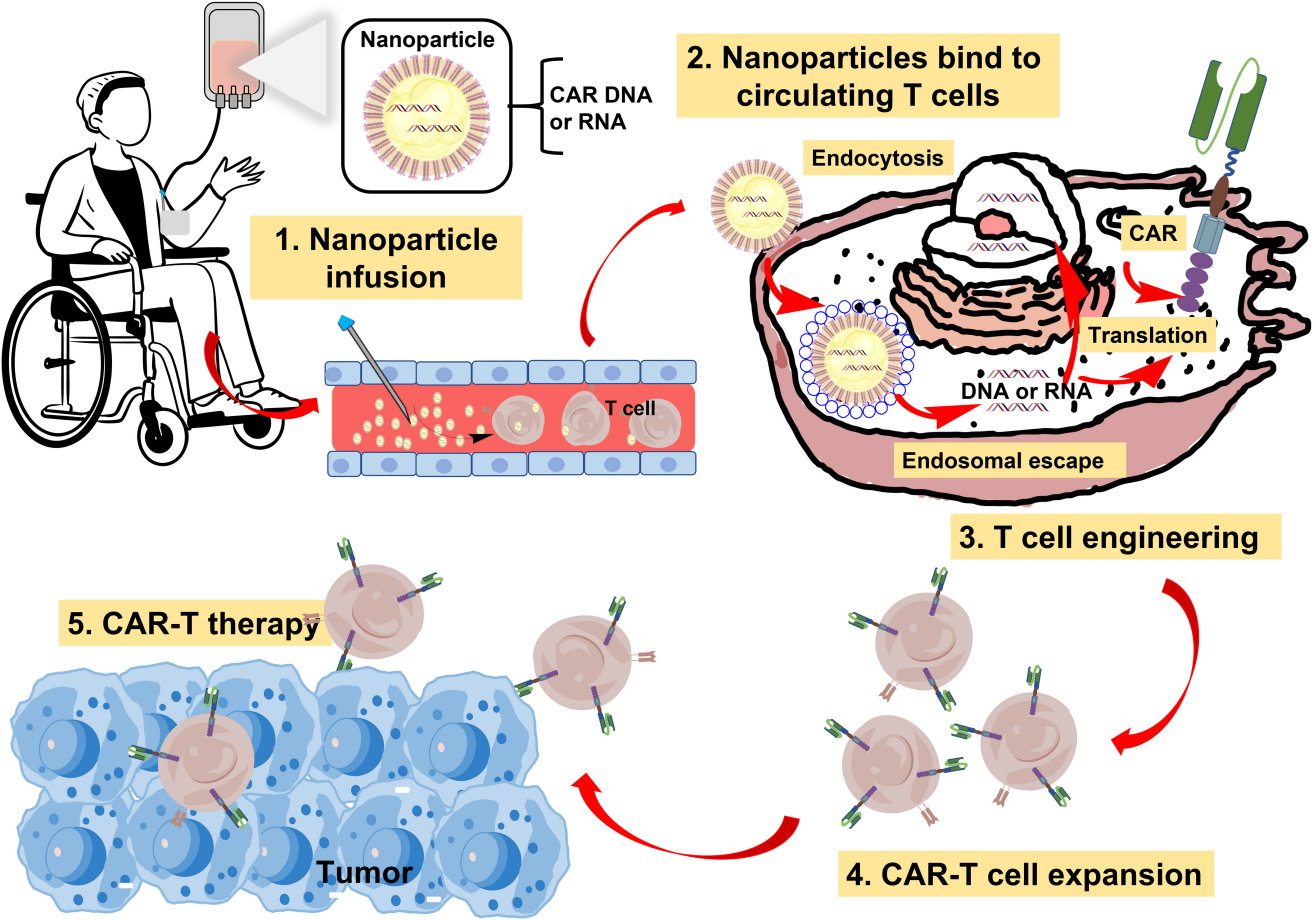
Schematic diagram of the process of *in-situ* CAT-T therapy with nanoparticles *in vivo*.

The conventional CAR-T therapy includes several steps, such as plasma apheresis from the patient, flow-sorting cells, CAR virus preparation, infection of CAR virus with T cells, expanded culture of CAR-T cells, and transfusion back to the patient after quality inspection (**Figure 2**) (18). It is not difficult to find that the process is extremely complex, and the laboratory conditions and operation requirements are very high, and the product is difficult to preserve. In addition, some shortcomings of viral vectors also limit the wide application of this method (19). Biomaterials can address the challenges of the above CAR-T therapy by providing economic and diversified gene vehicles (20). As gene vehicles, in addition to delivering genes to T cells *in vitro*, biomaterials can even rapidly and specifically program tumor recognition capabilities into T cells as they circulate *in vivo* (**Figure 3**). They are injected directly into the patient, after which the vehicles come into contact with the T cells, the T cell engineering, and the CAR-T cell expansion, and the treatment process occur spontaneously in the body (21). Compared with traditional methods, this reagent, called *in-situ* CAR-T cell programmed biomaterials, simplifies the process and avoids the step of extracting T cells to transform genes in non-primary environmental conditions. They can be easily mass-produced in a stable form, freeze-dried, stored, and used directly when needed. We cannot deny that directly generating CAR-T cells in patients is very challenging, but also the most clinically valuable. This is because it could transform CAR-T therapy from autologous cell-based drug products to generic, off-the-shelf drugs. With the rapid development of nanobiotechnology and gene editing technology, the latest research direction in this field is to enhance CAR-T cells and prepare them *in vivo* (22). The most common non-viral vehicles for gene delivery are cationic polymers and cationic liposomes. We will review the current research status of biomaterials as gene delivery vehicles in the generation of CAR-T cells, especially *in vivo*.

### 2.1 Cationic polymers for CAR gene delivery

Natural cationic polymers include chitosan, cyclodextrin, and their derivatives, while chemically synthesized cationic polymers contain poly-lysine (PLL), polyurethane (PU), polyethyleneimine (PEI), poly-L-histidine (PHSTD), and poly (2-dimethylaminoethyl methacrylate) (PDMAEMA), etc. (23). Cationic polymers can bind to negatively charged nucleotides (DNA, mRNA) to form complexes with unique conformation that protect the genes from enzymatic degradation, which improves the stability of the vector and nucleotide release. PEI is one of the fastest-growing gene vectors in recent years and has become the “gold standard” for gene delivery (24). Cationic polymers have been widely used for gene delivery since they were first proposed as carriers. To date, most cationic polymers have been able to overcome their non-degradable limitations by combining with a variety of degradable polymers to improve transfection efficiency and reduce biotoxicity (25).

Currently, researchers have developed various injectable *in-situ* CAR-T cell-programmed cationic polymers for the immunotherapy of tumors (**Table 2**). For example, Stephan et al. designed T cell-targeting cationic polymer nanoparticles for CAR DNA delivery in the treatment of leukemia (26). Positively charged nanoparticles were prepared by self-assembly of plasmid DNA expressing CAR with polymers combining nuclear localization signal peptides (NLS) and microtubule-associated sequences (MTAS). The above-obtained nanoparticles were further combined with polyglutamate (PGA-anti-CD3) of the T cell surface marker CD3 antibody to synthesize polymer nanoparticles for producing CAR in situ. The experimental results showed that the polymer nanoparticles were successfully prepared *in situ* for CAR-T cells in mice with leukemia and provided a long-term efficacy. *In vitro* transcription (IVT) mRNA is known for encoding therapeutic proteins in the cytoplasm, and its flexible and simple synthesis process enables cost-efficient production (34). The application of IVT mRNA is growing fast as it allows better control of the gene expression without risks of unexpected mutations caused by the genome integrations (35). More and more clinical trials and basic studies of CAR-T cells based on IVT mRNA have been reported (36). For example, in 2017 Stephan et al. tried a variety of cationic polymers to load CAR cargo, including hyperbranched polymers, polyethylene glycol-grafted polyethylene imine, and polymer (β-aminoester) (PBAE) polymers, which showed the possible use of polymerizing nanoparticles in mRNA-mediated CAR-T cell engineering (27). They found that biodegradable PBAE polymers were highly transferable and had minimal toxicity through mRNA transfection experiments. Besides, Pun et al. produced a specific pHEMA-g-pDMAEMA comb and sunflower cationic polymer used for DNA and mRNA delivery *in vitro* (28). These polymers can be transfected into Jurkat T cell lines with 25-50% efficiency in serum-free media. In 2020, Stephan et al. used PBAE to synthesize an mRNA nanocarrier for *in vivo* transient expression of circulating T cells (29). They mixed the mRNA of the transiently edited specific CAR or TCR with PBAE to prepare the positive-charged nanoparticles through self-assembly. Polyglutamate (PGA) carrying an anti-CD8 antibody was further modified on the above nanoparticles to confer its ability to target T cells to construct new *in-situ* CAR-T cell programmed nanoparticles. Repeated input of these mRNA polymer nanoparticles encoding CAR or TCR in orthotopic xenograft mouse models of lymphoma, prostate cancer, and hepatitis B-induced hepatocellular carcinoma could reprogram circulating T cells and thus induce an antitumor response. Because producing CAR-T cells *in vivo* is cost-effective, it has the potential to be an alternative to the current *in vitro* preparation protocol. Although some studies showed that the nanoparticles injected into mice were detected in non-T cells, the off-target risk can be addressed by conventional drug therapy (37).

**Table 2:** Nanomaterials for CAR-T cell generation.

Nanomaterials		Gene	Model	*In vitro* or *in vivo*
Nanomaterials for CAR gene delivery	**Cationic polymers**			
	Polyglutamate	DNA	Leukemia	*In vivo* (26)
	Polymer (β-aminoester)	mRNA	T lymphocytes	*In vitro* (27)
	pHEMA-g-pDMAEMA	DNA or mRNA	Jurkat human T cell line	*In vitro* (28)
	Polymer (β-aminoester)	mRNA	Leukemia	*In vivo* (29)
	**Cationic liposomes**			
	Imidazole-containing lipids	mRNA	T lymphocytes	*In vitro* and vivo (30)
	Ionizable liposome	mRNA	T lymphocytes	*In vitro* (31, 32)
Polydopamine nanoparticles for physical transfection of CAR-T cells		mRNA	Jurkat and human T cells	*In vitro* (33)

### 2.2 Cationic liposomes for CAR gene delivery

Since the first discovery that liposomes can deliver DNA to monkey kidney cells in 1980, it has become widely used as a non-viral gene vector (38). Liposomes are closed vesicles composed of hydrophobic groups, hydrophilic groups, and liposome linkers. The special structure makes liposomes less toxic, highly compatible, large compacity of target gene packaging, easy to prepare and modify, etc. (39). When genes are encapsulated in liposomes, it prevents the degradation by nucleases and promotes the escape of endosomes to effectively assist gene transfer. According to the different charges, liposomes are divided into cationic liposomes, neutral liposomes, and anionic liposomes (40). Cationic liposomes are one of the most commonly used non-viral gene delivery vehicles. Researchers have developed many approaches to improve the low transfection efficiency and targeting of cationic liposomes, including adding auxiliary materials, structural modification of the liposome surface, developing and designing novel lipid materials, and innovative preparation techniques (41). In addition, liposomes are combined with polymer nanoparticles to form lipid-polymer hybrid nanoparticles for *in vivo* and *in vitro* delivery of genes (42, 43). Last but not least, cationic liposomes are currently the most clinically and commercially available gene delivery vectors (44).

Recently, researchers used the cationic liposome-mRNA delivery system to enhance the auxiliary function of CAR-T cells and used it for the preparation of CAR-T cells *in vitro* and *in vivo*, which points the way for future development in the field. Xu et al. constructed a library of lipid-like nanoparticles and evaluated their role in delivering mRNA to T lymphocytes *in vitro* (30). Imidazole-containing lipids were identified from the library with the best transfection efficiency in mRNA delivery and were used for the efficient delivery of anti-CD19 mRNA CAR to human CD8^+^ T lymphocytes. In addition to that, Mitchell’s group prepared two libraries of ionizable liposome nanoparticles containing different alkyl chains and polyamine cores and selected the nanoparticles that could be used to efficiently deliver mRNA to CAR-T cells (31, 32). Comparing conventional electroporated CAR-T cells and the CD19 CAR-T cells treated with optimal liposomal nanoparticles, both approaches induced strong anti-tumor effects, but nanoparticle delivery significantly reduced the cytotoxicity. These results indicate that nanomaterials can deliver mRNA to T cells for CAR-T cell engineering and further immunotherapy. Taken together, these polymer nanoparticles are easily produced in a stable form, thus simplifying storage and reducing cost. Moreover, the process of nanocarrier preparation of CAR-T cells is greatly simplified compared with conventional lentivirus preparation procedures, which can provide a practical and widely applicable treatment that can generate anti-tumor immunity “on-demand” for patients in a variety of scenarios. The efficacy is comparable at the mouse level the use of nanoparticles to prepare CAR-T cells will hold greater promise.

In addition to viruses and nanoparticles as vehicles for transfection, physical transfection is another common approach for constructing CAR-T cells (45). Currently, electroporation is the most studied physical transfection technique used for the production of CAR-T cells with mRNA. However, conventional electroporation techniques are often associated with high levels of cell death, which is unfavorable due to the limited number of patient-derived T cells (46). Beyond this, the decreased function and dysregulation of gene expression of human T cells induced by electroperforation may reduce their antitumor activity (47). Thus, in the last decade, alternative physical transfection techniques considering both efficiency and safety were widely explored. Photoporation of nanoparticle-sensitization has been used as a more modest alternative to electroporation to transform T cells. Recently, Braeckmans’s team synthesized 0.5 µm polydopamine nanoparticles (PD NPs) coated with bovine serum albumin (BSA) as photoperforation sensitizers and successfully used them for physical transfection (33). PD NPs were mixed with cells and immediately subjected to laser irradiation to successfully form vapor bubbles that delivered FD500 and eGFP-encoded mRNA to HeLa cells and difficult-to-transfect Jurkat and human T cells, respectively. According to the results, the number of live transfected primary human T cells from nanoparticle photoperforation was about 2.5-fold higher than that from nuclear transfection. Given the advantage of the physical transfection of PD-BSA NPs, it is expected that they can be used to produce CAR-T cells.

## 3 Biomaterial-boosted CAR-T therapy in solid tumors

CAR-T therapy has shown limited efficacy in solid tumors. Similar to the delivery of other small molecule or biomacromolecule drugs, the treatment of CAR-T cells in solid tumors needs to undergo blood circulation and infiltrate into solid tumors to exert immunotherapeutic effects (48, 49). However, compared with hematological tumors, solid tumors are mostly characterized by the diversity of tumor cell targets, the inefficient infiltration of T cells, and a large number of immunosuppressive cells in the tumor microenvironment (TME) (50). Therefore, in the treatment of solid tumors, a single CAR-T therapy has a low response rate and a high recurrence rate. To improve the immunotherapeutic efficacy of CAR-T in solid tumors, multiple therapeutic strategies can be used simultaneously to cells orly attack cancer cells or improve the tumor microenvironment to promote the infiltration of CAR-T cells, or reduce immunosuppressive molecular signaling (51). Therefore, the development of various combination treatment strategies for CAR-T has become one of the main topics in this field.

**Figure 4:**
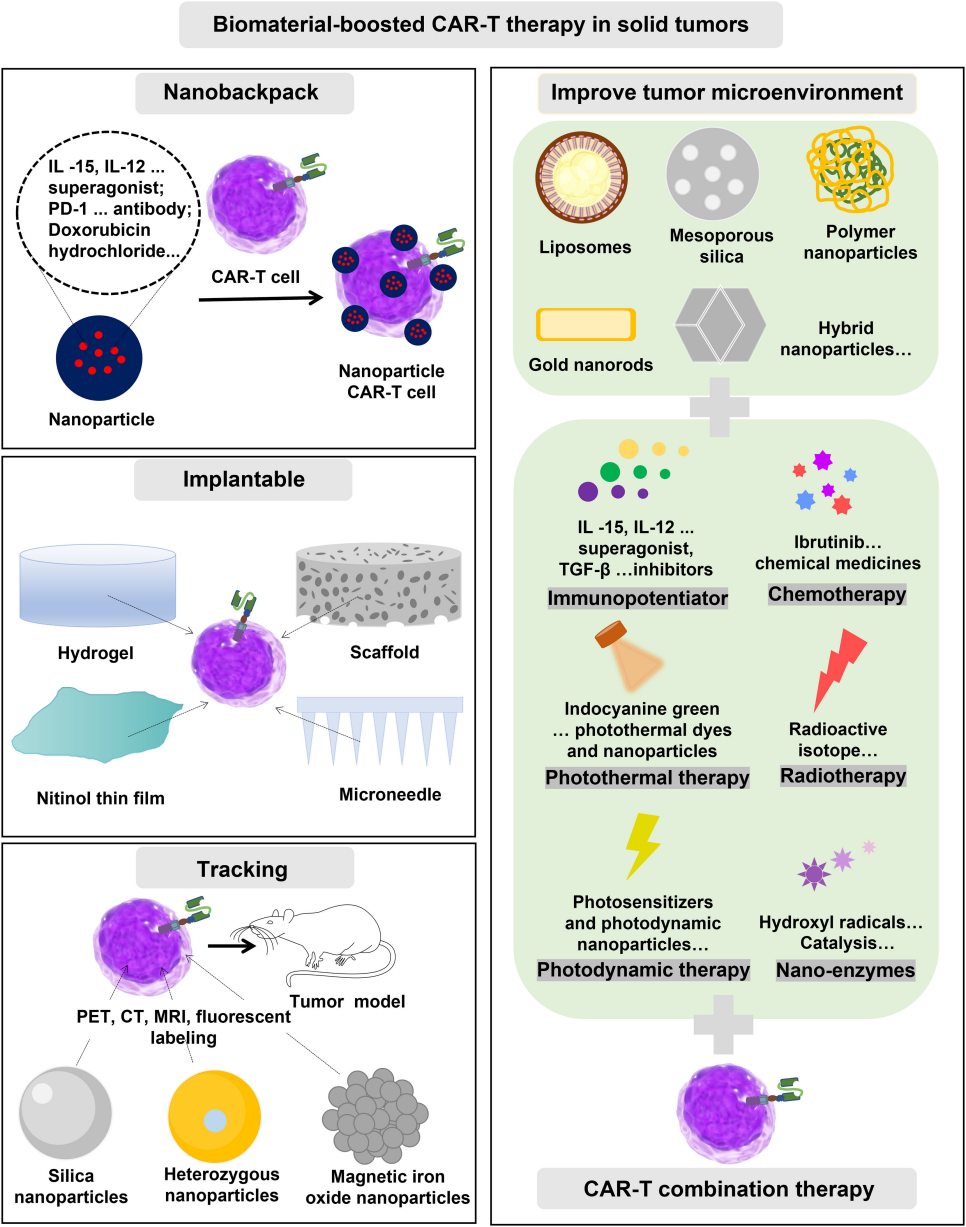
Several forms of biomaterials promote CAR-T therapy for solid tumors.

In recent years, emerging biomaterials have developed rapidly in oncology. Biomaterial-based drug delivery systems can utilize vascular abnormalities, hypoxia, or acidic microenvironments to induce therapeutic drugs to be released directly into the tumor environment, reducing off-target side effects. Novel biocompatible materials are flexible in structure and design, allowing them to be modified on cell surfaces or tailored for CAR-T combination therapy to improve immunotherapy efficacy (52–54). As is shown in **Figure 4**, it usually takes the following forms. First, in the backpack strategy of CAR-T therapy, before reinfusion, nanobackpacks can attach to CAR-T cells to support their return to the tumor site and directly and slowly release the carrying immune booster or other agents in the TME to enhance the effectiveness of therapy. Second, to target the enrichment of CAR-T cells at the tumor site to exert their immune effects, the implantable biomaterial was specifically designed to be loaded with CAR-T cells for local administration. Furthermore, microenvironment regulation plays a key role in the treatment of solid tumors, and the development of strategies to promote CAR-T cell infiltration based on improving the microenvironment is the key to enhancing the therapeutic effect. Various nanotechnology strategies, such as chemotherapy, phototherapy, radiotherapy, immune checkpoint blockade, and ultrasound therapy, have been used to modify TME and transform adverse tumor factors to facilitate immunotherapy. As shown in **Table 3**, we will summarize the research progress of CAR-T cells in the treatment of solid tumors.

**Table 3:** Biomaterial-boosted CAR-T therapy in solid tumors.

Nanomaterials	Cargo molecules	Model	Stage
Nanobackpack for CAR-T therapy			
Liposomal	IL -15 and IL-21 superagonist	Melanomas	Preclinical (55)
Protein nanogels	IL-2Fc, ALT-803, IL-15Sa superagonist	Melanoma	Preclinical (56)
HSA-based nanoparticles	IL-12 superagonist	Lymphatic tumor	Preclinical (57)
Liposome	A2a adenosine receptor antagonist	SKOV3.CD19 tumors	Preclinical (58)
Magnetic nanocluster	PD-1 antibody	Lymphoma and breast tumor	Preclinical (59)
Polylactide copolymers	Doxorubicin hydrochloride	Glioblastoma	Preclinical (60)
Implantable biomaterials for CAR-T therapy			
Macroporous scaffold	IL-15 superagonist	Breast and ovarian cancer	Preclinical (61)
Polysaccharide scaffolds	IL-15/IL15Rα cytokine	Pancreatic and melanoma	Preclinical (62)
Hyaluronic acid hydrogel	IL -15 superagonist and anti-PD-L1	Melanoma	Preclinical (63)
Fibrin-coated nitinol films	Antibodies against CD3, CD28 and CD137	Ovarian cancer	Preclinical (64)
Polymer-Nanoparticle hydrogels	IL-15 superagonist	Human medulloblastoma	Preclinical (65)
Alginate Scaffold	IL-12 superagonist	Lymphatic tumor	Preclinical (66)
Porous microneedle patch	No	Pancreatic and melanoma	Preclinical (67)
Biomaterials improve tumor microenvironment			
**Nanomaterials**	**Combination therapy**	**Model**	**Stage**
Liposomes	Immunopotentiator, TGF-β inhibitors	Melanomas	Preclinical (68)
Polylactic acid-glycolic acid	Photothermal, indocyanine green	Melanomas	Preclinical (69)
INPs nanoparticles	Photothermal, indocyanine green	Raji tumors	Preclinical (70)
FA-Gd-GERTs@Ibrutinib	Photothermal	Non-Hodgkin’s lymphoma	Preclinical (71)
Gold nanorods	Photothermal	K562 or Raji tumors	Preclinical (72)
Mesoporous silica	Photothermal, IR780	Liver cancer	Preclinical (73)
HA@Cu_2_−xS-PEG	Catalysis and photothermal	Non-small cell lung cancer	Preclinical (74)
Biomaterial tracking CAR-T cells in vivo			
**Nanomaterials**	**Imaging method**	**Model**	**Stage**
Silica nanoparticles	PET/NIRF	Ovarian peritoneal carcinomatosis	Preclinical (75)
GNP-64Cu/PEG2000	PET	Jurkat cells	Preclinical (76)
USPIOs	MRI	Glioblastoma	Preclinical (77)

### 3.1 Nanobackpack for CAR-T therapy

At present, traditional CAR-T therapy still has great limitations. The surface engineering of CAR-T using drug-loaded nanoparticles can improve the therapeutic effect. The researchers have tethered nanoparticles with different functions such as targeting, tracer, control, and regulation to T cells to construct a “backpack” system to enhance and improve the expansion and effectiveness of transduced T cells *in vivo* (78). For example, some immunomodulators can promote the immune response of CAR-T cells against the tumor, but the systemic administration has large toxic and side effects, so the targeted and local administration at the tumor or disease sites can improve the safety and efficacy of their treatment. In 2010, Irvine’s team constructed backpack CAR-T cells by attaching liposomal nanoparticles loaded with interleukin-15 (IL-15) and interleukin-21 (IL-21) superagonists to the cell membrane of CAR-T cells *via* maleimide thiol chemical coupling (55). In the treatment of tumors, the therapeutic efficacy of CAR-T is enhanced by “autocrine” stimulation of cytokines from the backpack. Subsequently, in 2018, the team developed a novel T-cell receptor-responsive nanogel (NGS) loaded with a large amount of IL-15 superagonist complex and conjugated it to the cell membrane surface of CAR-T for effective tumor clearance (56). In this process, since the “backpack” CAR-T cells do not release IL-15 superagonist complexes until they recognize tumor cells, this approach can effectively enhance tumor specificity and reduce the toxic side effects caused by off-target effects. The treatment results also demonstrated that the NGS delivery method selectively expanded T cells in the tumor 16-fold compared with the intravenous systemic administration of free cytokines. Moreover, in the B16F10 xenograft mouse model, the above administration resulted in complete tumor eradication in 80% of the mice. Recently, Cai’s team prepared HSA-based nanoparticles loaded with interleukin-12 (IL-12) combined with CAR-T cells for the treatment of solid tumors (57). IL-12 was loaded into nanoparticles (INS) by reassembling the internal disulfide bonds of HSA. INS-CAR-T backpack hybrids were prepared by copper-free click chemical reaction between dibenzocyclooctyl (DBCO)-modified INS and azide-labeled CAR-T cells. INS-CAR-T cells were injected intravenously into tumor-bearing mice, and IL-12 was released in response to high concentrations of glutathione in the solid tumor microenvironment. The results showed that IL-12 nanopacks significantly enhanced the efficacy of CAR-T cells in solid tumors. Wang et al., synthesized a cross-linked multilayer liposome (CML) nanoparticle loaded with A2a adenosine receptor (A2aR) antagonist SCH-58261 and further chemically conjugated it to the surface of CAR-T cells and used it for effective tumor treatment (58). In conclusion, immunomodulators loaded on nanopacks and then further loaded on the surface of CAR-T cells can effectively improve the therapeutic effect on solid tumors.

It is important to mention that immune checkpoint-blocking inhibitors and chemotherapeutic agents used in combination with CAR-T cells are also common and effective treatments. For example, Xie’s team constructed backpack CAR-T cells by binding magnetic nanocluster particles loaded with PD-1 antibodies to the surface of effector T cells overexpressing PD-1 through specific antibody-antigen interaction (59). Under the guidance of the external magnetic field, the effector T cells can be enriched to the tumor site, and at the same time, the hydrolysis of a benzoic acid-imine bond can release PD-1 antibody under the stimulation of the micro acid environment of the tumor, and play a synergistic role in killing the tumor with T cells. This study demonstrates that the combination of two core technologies, CAR-T therapy, and immune checkpoint inhibitor therapy, can improve the treatment of solid tumors. Besides, Dong et al. chemically coupled IL13-targeting nanoparticles loaded with doxorubicin hydrochloride (DOX) to the surface of CAR-T cells by click chemistry for glioblastoma treatment (60). In the tumor-bearing mouse animal model, the IL13-targeting of nanoparticles enriched both CAR-T cells and nanoparticles at the tumor site. CAR-T cells and DOX released from pH-sensitive nanoparticles in the stimulation of the tumor acid environment achieved synergistic treatment of the glioblastoma. Compared with free DOX, the above nanoparticle drug delivery method resulted in the accumulation of more DOX at the tumor site, and the accumulation of CAR-T cells in the tumor microenvironment was also increased. All these results demonstrate that the surface engineering of CAR-T cells by backpack nanoparticles has no effect on cell viability and function, and even provides a powerful tool for efficient and safe antitumor immunotherapy. In conclusion, the combination of nanomaterials and drug delivery technology to design backpack nanoparticles attached to the surface of CAR-T cells is a good strategy for combination therapy.

### 3.2 Implantable biomaterials for CAR-T therapy

CAR-T cells must be targeted to the tumor site to exert their therapeutic effects. However, the immunosuppressive microenvironment of solid tumors often prevents excellent targeting of CAR-T cells, resulting in poor therapeutic outcomes. Due to these obstacles, implantable biomaterials have been explored to form supportive delivery systems that can be loaded with CAR-T cells and overcome physical/physiological barriers to deliver CAR-T cells directly to the solid tumor site, promoting their sustained and local release while promoting orthotopic T cell proliferation (79). Thus, the tumor can be exposed to a high concentration of T cells for a long time to achieve sustained tumor killing.

The most representative is the implantable scaffold-type biomaterial delivery platform. For example, in 2015 Stephan et al. integrated collagen-mimicking peptides into a macroporous scaffold of sodium alginate as a delivery and release platform for CAR-T cells to improve the efficiency of tumor-targeted T cell metastasis (61). The scaffold was also loaded with mesoporous silica nanoparticles encapsulated with an IL-15 superagonist, which were coated with lipid bilayers containing anti-CD3, CD28, and CD137 antibodies. And the polymer allows nanoparticles and CAR-T cells to freely transfer, proliferate and spread. After the scaffold was implanted in the breast cancer excision site and the ovarian cancer tumor site, the continuous secretion of soluble IL-15 superagonist stimulated antibodies to maintain the “trigger” state of T cells, which effectively assisted the stable expansion and proliferation of CAR-T cells in the tumor area. The results showed that it effectively inhibited the growth of ovarian cancer and the recurrence of breast cancer after resection. As a control, tumor recurrence and death occurred in mice injected intravenously or intravenously with free T cells. The team then used the bioactive polymer to cooperatively deliver CAR-T cells and nanoparticles loaded with IL-15/IL15Rα cytokine to eliminate heterogeneous tumors. Tumor growth was effectively inhibited in both pancreatic cancer and melanoma *in-situ* mouse models (62).

In addition, hydrogel-type biomaterials have also been used to load CAR-T cells for tumor immunotherapy. Gu et al. simultaneously embedded antigen-specific CAR-T cells, IL-15-loaded PGLA nanoparticles, and anti-PD-L1 antibody-conjugated platelets in a biodegradable hyaluronic acid hydrogel to prevent postoperative tumor recurrence (63). The inflammatory microenvironment at the site of postoperative tumor resection can trigger platelets to produce platelet-derived particles (PMPs) to release anti-PD-L1 antibodies. At the same time, the sustained release of IL-15 agonists from PGLA nanoparticles maintained the “trigger” state of T cells to ensure the activation and proliferation of loaded CAR-T cells. In a mouse model of melanoma, this hydrogel local delivery platform resulted in longer sustained survival of CAR-T cells in the surgical space and significantly inhibited residual tumor growth compared with direct intratumoral or systemic administration of CAR-T cells and anti-PD-L1 antibodies. It should be noted that this local CAR-T cell immunotherapy also effectively inhibited distal tumor growth.

The above method of administration based on polymer scaffolds and hydrogels provides an effective platform for CAR-T cell immunotherapy for unresectable tumors or postoperative recurrence prevention. It is important to note that the pore structure of both polymer scaffolds and hydrogels is random, which makes the loading capacity and release kinetics of the cells not accurate and may ultimately affect the therapeutic effect of CAR-T cells. Therefore, the development of biomaterials with precise structures for loading CAR-T cells will help to improve the efficacy of immunotherapy. Nitinol (TFN) has been used to develop new medical devices because of its unique biological value. In addition, vacuum sputtering deposition technology has also been used to fabricate micropatterned Nitinol films with thicknesses ranging from a few microns to a few nanometers. Stephan’s team developed an implantable network of fibrin-coated nitinol films for the local delivery of CAR-T cells. When the film is used to culture CAR-T cells, the cells rapidly fill the lattice at a high density, with a pattern and number that can be predicted from the pore geometry of the material. In an unresectable ovarian cancer model, nitinol films loaded with CAR-T cells were implanted into the tumor site to promote the rapid expansion of T cells, resulting in direct delivery of a high density of T cells to the tumor, significantly improving survival in mice (64). Recently, Appel et al. prepared a polymer-nanoparticle hydrogel (PNP hydrogel) consisting of a dodecyl-modified hydroxypropyl methylcellulose polymer and biodegradable nanoparticles (65). CAR-T cells and IL-15 were mixed with PNP hydrogel and injected next to mouse tumors. The CAR-T cells activated and multiplied in response to IL-15 and were released from the hydrogel to kill the cancer cells. After 12 days, the mice’s tumors were eliminated. In addition, the above treatment can also eliminate the distal tumor. Brudno et al. developed a multifunctional alginate scaffold (MASTER) for T cell programming and release, which was validated in the treatment of mouse lymphoma (66). MASTER therapy showed superior results compared to conventional CAR-T therapy. Besides, Gu’s team developed a biocompatible poly (lactate-glycolic acid) (PLGA) porous microneedle patch loaded with CAR-T cells and implanted into the tumor bed or postoperative excision cavity for *in-situ* penetration-mediated immune cell seeding to inhibit tumor growth and recurrence (67). Due to the presence of microneedle tip pores, loaded CAR-T cells can be uniformly dispersed into the tumor without loss of activity. This microneedle-mediated local delivery significantly inhibited tumor recurrence and growth by enhancing CAR-T cell infiltration and immune stimulation compared with direct intratumoral injection in postoperative resected melanoma and orthotopic pancreatic tumor models. To sum up, implantable biomaterials provide a new research method for CAR-T cell immunotherapy and deserve extensive promotion.

### 3.3 Biomaterials improve tumor microenvironment to enhance CAR-T therapy

Numerous studies have shown that single CAR-T therapy for the treatment of solid tumors still has significant limitations. Synergistic therapy combined with chemotherapy, photothermal therapy, metabolic regulation, or other immunotherapy has attracted increasing attention, which is expected to overcome each other’s shortcomings and achieve satisfactory treatment results. The following is a review of recent research on the ways nanoparticles have been used to reshape the tumor immune microenvironment to facilitate CAR-T therapy, including specifically eliminating immune suppressor cells, reprogramming immune regulatory cells, promoting inflammatory cytokines, and blocking immune checkpoints.

The immunopotentiator can restore the low immune function to normal or has the adjuvant function, enhance the antigen’s immunogenicity combined with it, and accelerate the induction of immune response (80, 81). Or it can replace the lack of immune active ingredients in the body, the immune replacement effect. In addition, it may also have a bidirectional regulation effect on the immune function of the body, so that the immune function tends to be normal when it is too high or too low. Therefore, researchers have made some progress in combining immunopotentiators with CAR-T cells to enhance the antitumor efficacy of immunotherapy. Many nanobackpacks and implantable biomaterials promoting CAR-T therapy also involve the combination of immunopotentiators, which have been summarized previously. Furthermore, Irvine et al. prepared pegylated immunoliposomes carrying TGF-β inhibitors targeting CD45 to enhance CAR-T therapy. In the B16F10 xenograft tumor-bearing mouse animal model, the immunoliposome was injected before *in vivo* injection of CAR-T cells, which inhibited tumor growth (68). Thus, the injection of nanoparticles loaded with immunopotentiators to modulate the microenvironment before the injection of CAR-T cells is also a good therapeutic approach.

Chemotherapy drugs can reshape the TME by selectively suppressing the activity of immunosuppressive cells, such as Treg cells and MDSCs (82). Therefore, the combination of CAR-T cells with chemotherapy agents may be a promising strategy to improve the antitumor efficacy of CAR-T cell immunotherapy. Wolf’s team designed new CAR-T cells that target prostate cancer antigen (PSMA) recognition and combined them with low doses of docetaxel to treat prostate cancer (83). In a prostate cancer xenograft model, local injection of PSMA CAR-T cells eradicated tumors, and intravenous injection of PSMA CAR-T cells significantly inhibited tumor growth. However, neither CAR-T cells nor drugs alone had a therapeutic effect. These results indicate that the combination of chemotherapy and CAR-T cells is a promising approach to cancer treatment. Although the study was designed to promote CAR-T therapy by injecting docetaxel directly into the body. However, because of our understanding of the preponderance of nanomaterials as drug delivery vehicles, we firmly believe that coating drugs in nanomaterials will lead to better therapeutic effects and avoid unnecessary systemic side effects.

Radiotherapy and photodynamic therapy can not only kill tumor cells directly but also lead to the release of proinflammatory cytokines and chemokines to regulate the tumor microenvironment (84, 85). Therefore, the combination of CAR-T with them can further improve the efficacy of cellular immunotherapy. Roth et al. combined radiotherapy with CAR-T cells expressing NKG2D (NKG2-DII type integrated membrane protein) for the synergistic treatment of murine glioblastoma (86). The experimental results show that this method can effectively prolong the survival time of mice carrying *in-situ* glioblastoma. Sadelain et al. combined low-dose sensitized radiation with CAR-T cells for the treatment of heterogenous orthotopic pancreatic cancer (87). And the results indicate that CAR-T cells alone cannot eradicate these heterogeneous tumors. But, synergistic therapy can overcome antigen escape and effectively eliminate tumors with heterogeneous tumor-associated antigen expression. Although the above two examples do not involve biomaterials, it is not difficult to believe that the design of nanomaterials to improve the efficacy of radiation therapy is an effective way of radiation-enhanced CAR-T therapy.

Photothermal therapy is a treatment method in which materials with high photothermal conversion efficiency are injected into the body, gathered near tumor tissues by targeted recognition technology, and converted into heat energy to kill cancer cells under the irradiation of external light sources (generally near-infrared light) (88, 89). Photothermal therapy offers unique advantages over conventional cancer therapies, including high selectivity, low systemic toxicity, and limited therapeutic resistance (90). Mild hyperthermia of tumors can reduce their dense structure and interstitial fluid pressure, increase blood perfusion, release antigens, and promote the recruitment of endogenous immune cells (91). Thus, the combination of mild hyperthermia with the adoptive transfer of CAR-T cells would increase the therapeutic index of these cells in solid tumors. The Gu’s team combined photothermal therapy with CAR-T to achieve effective therapeutic effects (69). In nod scid gamma (NSG) mice implanted with human melanoma WM115 tumors, polylactic acid-glycolic acid (PLGA) nanoparticles loaded with indocyanine green (ICG), a photothermal NIR dye, were first injected into the tumors. An 808 nm laser was then applied to illuminate the tumor to gently heat the solid tumor. Then, CAR-T cells targeting the antigen chondroitin sulfate proteoglycan-4 (CSPG4) were infused intravenously to achieve synergistic photothermal and immunological treatment. In this process, mild tumor heating can directly kill tumor cells, but also partially destroy them or the extracellular matrix, thereby reducing the density of solid tumors and high interstitial fluid pressure (IFP), dilating tumor blood vessels, and thereby leading to increased infiltration and accumulation of CAR.CSPG4^+^ T inside the tumor. Cai’s team synthesized nano-photosensitizer-engineered CAR-T biohybrids (CT-INPs) for the immunotherapy of solid tumors (70). They constructed CT-INPs by coupling nanoparticles loaded with indocyanine green photosensitizer to CAR-T cells *via* copper-free click chemistry. Under laser irradiation, CT-INPs showed mild photothermal effects to reshape the tumor microenvironment (disruption of extracellular matrix, dilation of blood vessels, relaxation of dense tissue, and stimulation of chemokine secretion), increase the infiltration of CT-INPs, and preserve the original activity and function of CAR-T cells. The photothermal effect induced by CT-INPs effectively breaks down the physical and immune barriers of solid tumors and strongly promotes CAR-T immunotherapy. Ye’s team synthesized multifunctional therapeutic nanoparticles (FA-Gd-GERTs@Ibrutinib) in combination with CD19 CAR-T cells for multimodal imaging and treatment of Non-Hodgkin’s lymphoma (NHL) (71). Nanoparticles were injected into tumor-bearing mice and enriched at the tumor site under FA targeting. When the tumor was irradiated with an 808nm laser, the temperature effect of nanoparticles improved the tumor microenvironment while killing cancer cells and increasing the infiltration of CD19 CAR-T cells, thus enhancing the treatment. In addition, Kwong et al. designed an engineered heat-specific gene switch and introduced it into CAR-T cells for tumor-specific therapy in combination with the photothermal effects of gold nanorods. In a tumor-bearing mouse model, gold nanorods were injected intravenously into mice before CAR-T cells were injected (72). Tumor temperature is slightly increased by local laser irradiation, and this thermal effect triggers the expression of engineered heat-specific genes in tumor-site CAR-T cells, enhancing antitumor activity while alleviating antigen escape. Yuan’s team has combined a nano drug (CIMs) delivery system with CAR-T cell targeting for the treatment of liver cancer (HCC) (73). These CIMs nanoparticles were constructed by coating the membrane of CAR-T cells that specifically recognize GPC3^+^ HCC on the surface of IR780-loaded mesoporous silica nanoparticles. The nanoparticles were injected into tumor-bearing mice to achieve effective tumor enrichment by targeting the cell membrane of CAR-T and to achieve photothermal treatment of tumors under 808nm laser irradiation.

It is important to mention that nano-enzymes have also been used to improve the tumor microenvironment and enhance CAR-T’s immunotherapeutic effect. Zhao et al. developed a tumor-targeting HA@Cu_2_-xS-PEG (PHCN) nanoenzyme and combined it with CAR-T cells for the treatment of non-small cell lung cancer (NSCLC) (74). The nanoenzyme was injected into the body and targeted to enrich the tumor site. Hydroxyl radicals generated by catalysis and photothermal effects generated by laser irradiation improve the tumor microenvironment while killing tumor cells, thereby enhancing the therapeutic efficacy of CAR-T cells targeting B7-H3. In summary, previous studies have shown that the synergistic effect of CAR-T and other therapies, especially the improvement of tumor microenvironment, can improve the immunotherapeutic effect of solid tumors.

### 3.4 Biomaterial tracking CAR-T cells *in vivo*

CAR-T therapy is often accompanied by adverse effects, such as off-target recognition of the wrong site by transfused immune cells, neurotoxicity, and cytokine release syndrome. Therefore, the safety of CAR-T therapy has always been the focus of attention. *In vivo* cell tracking technology can help us to better monitor the *in vivo* distribution, infiltration, persistence, and therapeutic efficacy of CAR-T cells (92). Effective and durable imaging techniques to track the distribution and fate of CAR-T cells *in vivo* will help us better understand the biological process of T cells in cancer therapy (93). This allows researchers to better adjust injection methods and evaluate doses to improve treatment effectiveness while avoiding potentially fatal systemic toxicity.

In the past, CAR-T therapy in solid tumors has been monitored mainly by measuring tumor size and morphological changes such as CT scan/magnetic resonance imaging (MRI) (94, 95). These methods can provide information about the tumor, but it is difficult to detect the spatial information of the injected T cells. However, with the development of molecular biology and medical imaging technology, it is possible to visualize biological processes (96, 97). Bioluminescence and fluorescence imaging have high sensitivity but lack tomography information and poor tissue penetration (98, 99). MRI has high spatial resolution but relatively low sensitivity (100). The combination of radionuclide imaging with CT or MRI is highly sensitive and has become the most frequently used technique in clinical practice (101, 102). It can be seen that different imaging techniques have different characteristics. With the rapid development of nanomaterials in imaging, many researchers are combining them with the above imaging techniques to construct non-invasive cell-marker contrast agents and use them to reveal the biological distribution of CAR-T cells in the body. For example, the Aras’ team developed an artificial heterozygous CAR-T imaging platform based on dual-mode positron emission tomography (PET) and near-infrared fluorescence (NIRF) (75). CAR-T cells were nongenetically labeled *in vitro* using ^89^Zr and near-infrared fluorescently labeled silica nanoparticles. Then, it was injected into Vivo, and PET/NIRF imaging was used to monitor the biodistribution of CAR-T *in vivo*, which guides the evaluation of the therapeutic mode of CAR-T therapy in solid tumors. Cooper et al. prepared heterozygous nanoparticles (GNP-64Cu/PEG2000) using macrocyclic chelators, polyethylene glycol, and ^64^Cu^2+^-labeled gold nanoparticles (76). GNP-^64^Cu/PEG2000 was electroporated into transgenic CD19-targeted CAR-T cells *via* the sleeping beauty transposon/transposase system. The CAR-T cells were injected into mice and their distribution *in vivo* was examined by PET imaging. Zhang et al. synthesized ultra-small superparamagnetic iron oxide nanoparticles (USPIOs) coated with amino alcohol derivatives of glucose for noninvasive monitoring of dynamic infiltration and persistence in glioblastoma (GBM) (77). All these studies demonstrate the feasibility of nanomaterial-based CAR-T cell direct labeling *in vitro* and imaging *in vivo*. However, defects such as signal dilution caused by cell division or death still limit its further clinical transformation, which needs to be further studied to find a solution. It is believed that this problem can be overcome by designing unique biomaterials to enhance the signal.

## 4 Challenge and outlook

With explosive clinical advances in the field of cancer immunotherapy, CAR-engineered T cells have become a mainstream treatment strategy. Biomaterials provide a broad and versatile tool library for their development to overcome many of the limitations. However, many challenges remain in the process of biomaterials promoting CAR-T therapy. During the generation of CAR-T cells, nanomaterials can serve as vehicles for CAR gene delivery to T cells and can also enable *in-situ* preparation of CAR-T cells *in vivo*. It has to be mentioned that this *in vivo* CAR gene delivery strategy may bring about undesired gene delivery to non-targeted cells. Moreover, improving the transfection efficiency of nanomaterials as CAR gene vehicles is also a key issue. Compared with the CAR-T cells prepared by viral vehicles, which have been approved by the FDA for clinical, the interaction of nanomaterials and the immune system is still unclear and controversial. Besides, although the treatment of CAR-T application in solid tumors is developing, its progress is relatively slow. The main reasons for the poor efficacy of CAR-T in solid tumors are heterogeneity, the lack of specific targets, the tumor immunosuppression microenvironment, and the multiple difficulties of the homing and colonization of CAR-T cells. As mentioned, biomaterials can enhance the efficacy of CAR-T therapy for solid tumors by improving the tumor microenvironment. But it is well known that nanomaterials for cancer treatment development still have disadvantages such as low targeting efficiency, poor tumor penetration, and obvious side effects, which also exist in CAR-T treatment. In general, further efforts are needed to improve its role in CAR-T treatment by designing biomaterials with special properties.

In addition, the safety and efficacy of biomaterials in CAR-T therapy have proven to be critical due to their complex composition. According to the record, one of the main features of US FDA-approved nanomaterials-related drugs is their simple construction and ease of repeat preparation. However, most of the nanomaterials used for CAR-T treatment are extremely complex in design and contain a wide variety of chemical or bioactive components, so it is also more difficult to regulate their quality and efficacy. It is conceivable that the more complex the biomaterials construct, the more difficult mass production on a clinical scale. The biomaterials used as delivery systems may continuously interact with the loaded CAR-T cells, so their clinical evaluation is highly demanding. This also leads to the extremely challenging use of biomaterials for clinical treatment. Currently, a high-throughput barcode screening technology based on next-generation sequencing (NGS) has been used to effectively and accurately identify new materials that can target specific cells or tissues (103). This will also provide the possibility of providing tailored biomaterials for CAR-T therapy. Therefore, in the initial stage of designing biomaterials for *in vivo* therapy, the possibility of future clinical application should be considered, to fundamentally investigate the cost, reproducibility, and the possibility of scale production.

## 5 Conclusion

**Figure 5:**
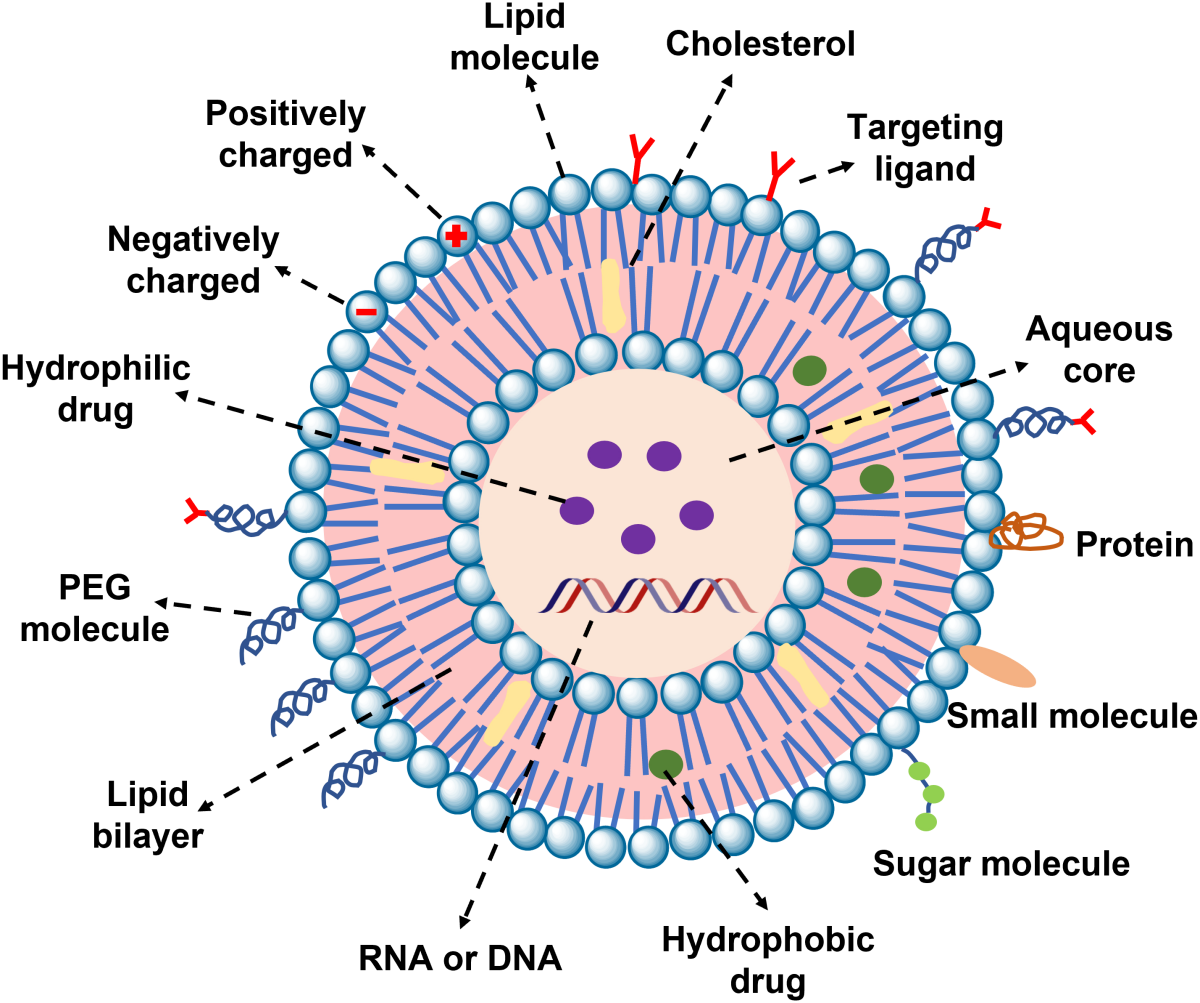
The Schematic diagram of liposomes and their multiple surface functionalization.

**Figure 6:**
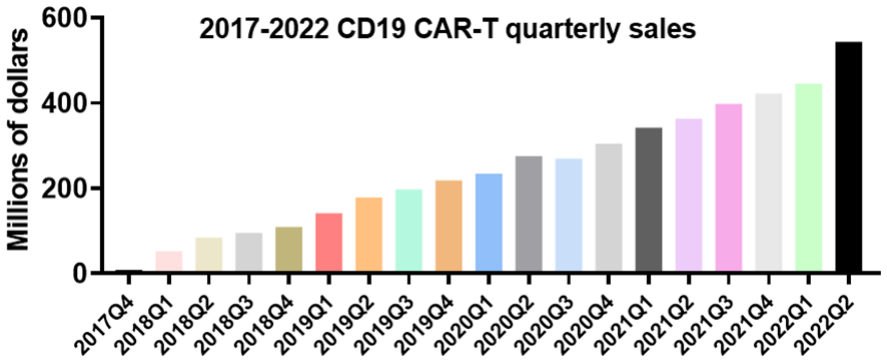
Sales of CD19 CAR-T products in each quarter between 2017 and 2022.

In summary, despite the encouraging progress of biomaterials for enhancing CAR-T immunotherapy. But there are still many obstacles to tailoring the inherent properties of immune cells and providing them with auxiliary functions. The details of the biomaterials used for CAR-T therapy need to be further elucidated. These insights will provide important guidelines for the development of safe and inexpensive biomaterials to enhance immunotherapy. It is important to be emphasized that all aspects of biomaterial design should be carefully considered to meet their applications. For example, the complex composition and physical properties of liposome nanoparticles, such as size, morphology,

surface charge, loaded with drugs or genes, ligand type, and density can change the characteristics of CAR-T cells unexpectedly (**Figure 5**). Overall, immunologists, oncologists, and materials experts must work closely to strengthen the establishment of interdisciplinary disciplines and accelerate potential clinical translation. This cross-sectional study between disciplines creates tremendous opportunities to understand the clinic and technology of new cancer therapies. In addition, it can be seen from the “Research Report on the Development Trend Forecast and Investment Strategy of CAR T Cell Therapy Industry in China” published by Huajing Industrial Research Institute that the relevant products already in clinical use are all for the treatment of hematologic tumors, and the sales volume is increasing year by year (**Figure 6**). There is still a large market space for CAR-T clinical therapy, especially for solid tumors. According to statistics, there have been dozens of liposome nanocarriers in clinical trials, which has attracted much attention. We believe that the combination of biomaterials and CAR-T will be a new era in the development of cellular immunotherapy.

## Author contributions

This review paper was overseen and revised by W-YL, XW, X-WH, and Y-KZ. Y-TQ and Y-PL were involved in data collection, literature review and manuscript drafting. All authors contributed to this article and approved the version submitted.
